# Direct observation of shockwaves, cavitation, and jetting inside liquid droplets during high-speed impact

**DOI:** 10.1016/j.ultsonch.2026.108121

**Published:** 2026-10-17

**Authors:** Vid Agrež, Silvestre Roberto Gonzalez-Avila, Darja Horvat, Run-Ze Xie, Qingyun Zeng, Rok Petkovšek, Claus-Dieter Ohl

**Affiliations:** a Department of Soft Matter, Institute for Physics, Otto-von-Guericke University, Magdeburg 39106, Germany; b University of Ljubljana, Faculty of Mechanical Engineering, Aškerčeva 6, Ljubljana 1000, Slovenia; c Insight Ontario, Inc. Coquitlam, BC V3J 0E8, Canada; d College of Shipbuilding Engineering, Harbin Engineering University, Harbin 150001, China; e Sanya Nanhai Innovation and Development Base of Harbin Engineering University, Sanya 572024, China

**Keywords:** Cavitation, Droplet impact, Shockwaves, Acoustic focusing, High-speed imaging

## Abstract

This study reports direct experimental observations of shockwave propagation and cavitation bubble formation inside liquid water droplets during high-speed impact (80 –180 m/s) on solid surfaces, together with jetting observed at the distal end of the droplet. Ultra-high-speed shadowgraph and angle-view imaging at 10 million frames per second resolved two distinct internal wave structures: a primary compression front (1500–2050 m/s), consistent with pressure-dependent corrections to the acoustic wave speed, and a secondary tension wave emerging approximately 500 ns later, generated by reflection at the curved water–air interface. The curved distal surface focuses converging rarefaction waves into a caustic region, leading to cavitation nucleation 1.2–1.5 µs after impact. The subsequent asymmetric collapse of these cavitation structures drives directed jetting from the distal end, with jet morphology dependent on droplet geometry: coherent jets for smaller spherical droplets (<2 mm) whereas spray-like ejection is typical for larger, elongated droplets (>2 mm). At the proximal end of the droplet, air is entrapped between the surface and the droplet, collapsing into a toroidal-shaped bubble that exhibits oscillations in the 0.5–1.8 MHz range. All presented results, including experimental data and accompanying analysis, provide benchmark validation data for computational models of compressible droplet impact.

## Graphical abstract

**Figure:**
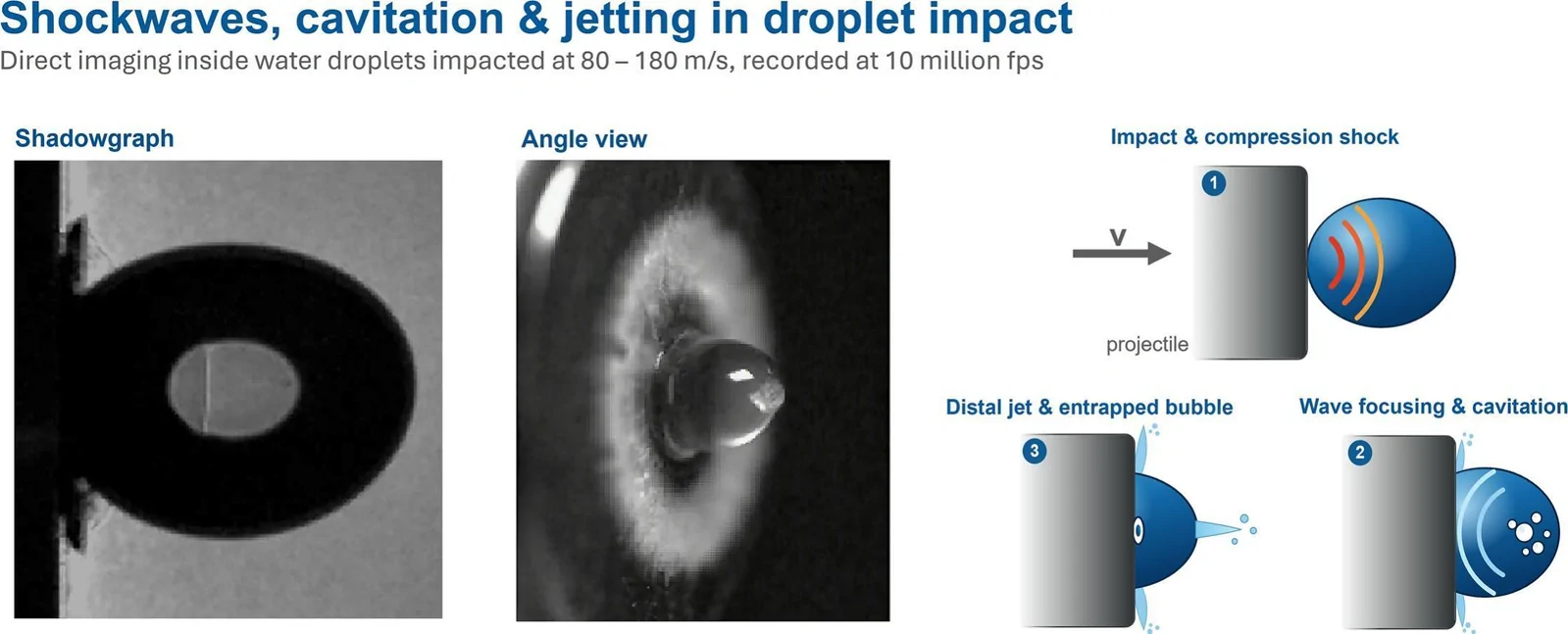

## 1 Introduction

The impact of a liquid droplet on a solid surface is a complex and highly dynamic process with significant relevance across many fields. High-speed impacts exceeding 100 m/s are particularly important in applications where erosion, splashing, or rapid phase change are critical, including transportation (aviation, high-speed trains, spacecraft re-entry), wind and steam/gas turbines, combustion and fuel injection, inkjet printing, spray cooling, and materials testing. Characteristic impact velocities vary considerably across these applications: aviation and aerospace systems experience impacts at 200–1500 m/s affecting radomes and windshields, steam turbines operate with blade leading edge impacts at 400–900 m/s and wind turbines experience blade leading edge erosion at 50–140 m/s [1]. When a droplet strikes a solid surface, it undergoes rapid deformation influenced by inertial, viscous and surface tension forces. The localized, transient high-pressure regions that arise due to the sudden deceleration of the droplet upon impact initiate many dynamic phenomena, including lateral jetting at the droplet–solid interface, shock wave generation and propagation within the liquid, through the ambient gas and the impacted solid, followed by wave reflection inside the droplet, resulting in acoustic focusing and bubble formation along caustic regions, possibly leading to jetting from the distal tip of the droplet. Because these events occur on extremely short timescales, capturing these phenomena requires advanced high-speed experimental techniques with precise temporal and spatial resolution. Such experiments remain scarce even today, particularly for high-speed impact scenarios.

Droplet impact dynamics have long been a subject of research, with foundational work by Worthington [2], characterizing the spreading and splashing of droplets on solid surfaces, and reviews throughout the years describing the evolution of the field [3], [4], [5], [6], [7], [8], [9], [10], [11], [12], [13]. A basic analytical model was proposed in [14].

Direct, time-resolved measurement of internal pressure fields or propagating waves within a millimetre-scale droplet during impact is prohibitively difficult, as the event unfolds over microsecond timescales and sub-millimetre spatial scales. Experimental studies that typically rely on high-speed optical techniques such as shadowgraphy and schlieren, visualize density gradients and shock fronts, while post-impact cavitation bubbles or fracture patterns serve as proxies for transient negative pressures. Most available experimental data concern low-speed impact (free falling droplets), while very few involve impact velocities exceeding 10 m/s, i. e. up to 50 m/s at most [15], [16]. Notable exceptions include Lesser et al. [3] who reported impact velocities up to 750 m/s, but using water jet tips; Field et al. [17], who achieved up to 300 m/s with two-dimensional gelatin drops and recent data, reported by Gonzalez-Avila et al. [18] who investigated 70–245 m/s impacts. The data was utilized in simulations by Nykteri et al. [19]. Consequently, many modeling and numerical approaches continue to rely on experimental results obtained more than four decades ago [8], [20], [21]. The scarcity of experimental data at impact velocities relevant for erosion has also been pointed out recently [9], [13], [15], [22], [23]. Numerous contributions to modelling and simulation of pressure fields inside and near impacted droplets, including the subsequent formation and evolution of shockwaves, cavitation, jetting, and proximal bubble entrapment, have been reported. Theoretical and numerical investigations predicting negative pressure in the distal region of the droplet, leading to bubble formation, can be found in [24], [25], [26], [27], [28]. More recently, Biasiori-Poulanges et al. [23] described this phenomenology in detail, comparing numerical predictions with the experiments of [17], both of which were performed on cylindrical droplets. Cavitation inside impacted liquid volumes was proposed as a possible damage mechanism already by Obreschkow et al. [24], and its effect on erosion damage thresholds was investigated by Field et al. [20]; nevertheless, it has received comparatively little attention in models of liquid impingement erosion. Very recently, Hao et al. [29] showed numerically that cavitation generated by wave reflections inside an impacting raindrop can substantially increase the stresses experienced by a wind turbine blade coating, suggesting that cavitation may contribute to rain erosion more than assumed in previous models. Related phenomena have also been investigated in the context of external shockwave impact on droplets [23], [30], [31], [32]. Proximal bubbles and air entrapment at the droplet–solid interface, observed already in [17], were reported at low impact velocities (up to 10 m/s) by Thoroddsen et al. [33], [34], Langley et al. [35], Lee et al. [36] and Liu et al. [37]. Further experimental confirmation was provided by Foltyn et al. [38]. At higher impact velocities, bubble formation at the wall surface was reported in [18], who regarding this observation pointed to the theoretical models of Hicks et al. [39], [40]. Direct measurements of air layer profiles under impacting droplets, using high-speed colour interferometry, were carried out by Van Der Veen et al. [41]. Other notable contributions to the development of analytical and computational tools to quantify droplet impact outcomes by modelling and simulations are reported in the literature [22], [42], [43], [44], [45], [46], [47], [48], [49], [50], [51].

The present study focuses on the observation of shock formation and phase change within the droplet during high-speed impact. Using a high-speed camera with 100 ns interframe time, in combination with a custom-designed synchronized pulsed illumination, we investigate the impact of liquid water droplet onto solid projectiles at velocities ranging from 80–180 m/s. The experimental investigation employs combined shadowgraph and angle-view recording techniques to visualize the phenomena within liquid droplets. This approach enables direct optical observation and characterization of shockwave propagation, cavitation bubble nucleation and subsequent jet formation at the distal end. Entrapped air bubble dynamics at the proximal interface are captured through light intensity fluctuation measurements. This work represents a direct experimental observation of shockwaves inside impacting 3D liquid droplets, extending the classical two-dimensional gelatin studies from Cavendish laboratories [17]. These findings provide data for validating computational models of compressible droplet impact. The present experiments do not address the response of the impacted solid; however, direct evidence of the phenomena occurring inside the droplet is a prerequisite for studying the loading that they may impose on the surface in the applications mentioned above.

## 2 Methodology

### 2.1 Experimental setup

A high-speed droplet impact experimental setup was developed to investigate collision dynamics between solid projectiles and acoustically levitated water droplets (Fig. 1) at velocities ranging from 80 m/s to 180 m/s. The experimental system comprises a projectile launcher consisting of a steel pipe (2.0 m length, 12.0 mm inner diameter) connected to a high-pressure nitrogen supply through a ball valve assembly, with operating pressures from 5 to 60 bar, generating the specified velocity range. A PTFE sleeve provides pneumatic sealing at the pipe base.

**Fig. 1:**
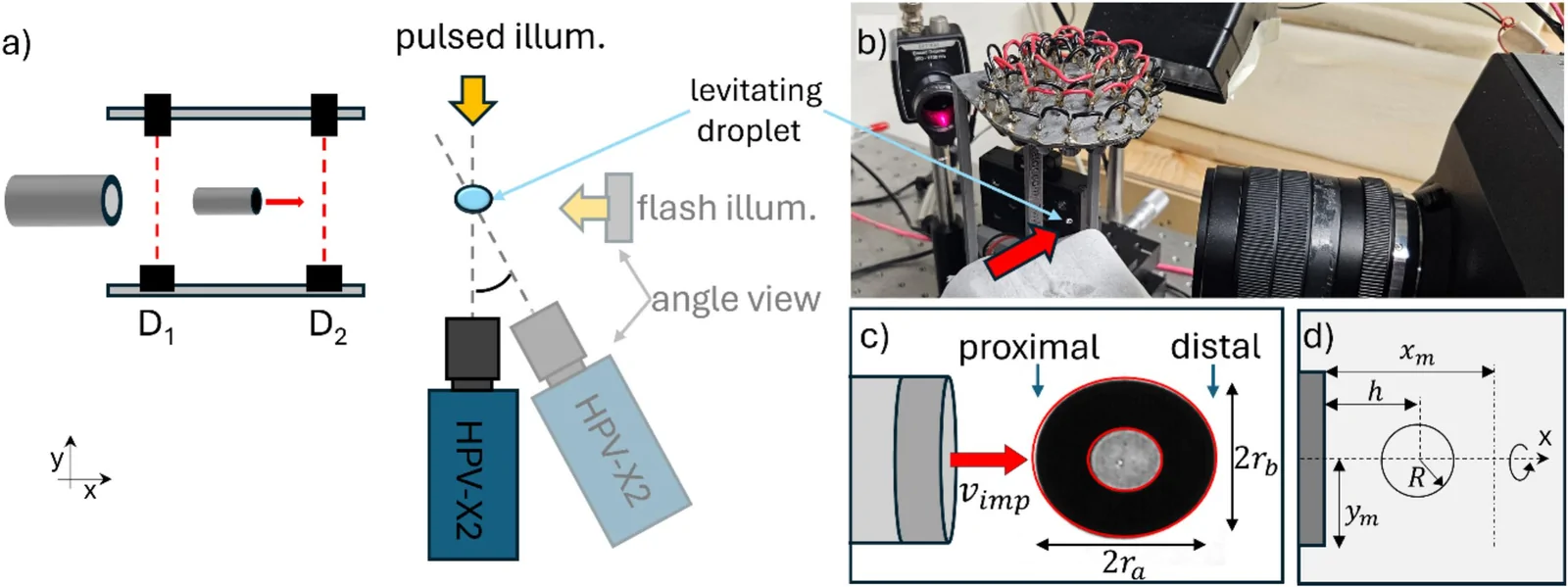
a) Experimental setup. b) Image of a water droplet inside the acoustic levitator, c) impact geometry and illustration of the visible window within the droplet (small gray circle), d) schematic diagram of the numerical simulation geometry. to determine actual dimensions inside the droplet, the measurements were corrected for refraction.

The projectiles were fabricated from PTFE having stainless steel impacting surfaces (6.6 g, 41.2 mm length), featuring 11.9 mm diameter with a 6 mm solid front section and hollowed rear section (3 mm wall thickness) to optimize weight distribution.

Water droplets were generated using a needle and positioned with an acoustic levitator that produced a standing wave field. Precise alignment was achieved using a three-axis positioning stage coupled with alignment laser beam. The droplets were elongated and can be well approximated by an ellipsoid, with the major axis aligned with the projectile velocity (Fig. 1c). The droplet major-axis diameters *2r_a_* ranged from 1.40–3.16 mm, and the median aspect ratio of the axes (*r_a_* / *r_b_*, major to minor) was 1.27 ± 0.16. The region of the droplet that first contacts the projectile is referred to as the proximal region, whereas the opposite region is termed distal. The droplet size is defined as the major axis diameter (*2r_a_*).

Triggering synchronization was achieved through a light gate consisting of a red LED and photodetector positioned at the barrel exit, where the photodetector signal activated the oscilloscope, which in turn controlled the digital delay generator for camera operation and illumination timing.

Projectile recovery and safety containment were ensured by multiple foam layers extending to a total thickness of 1 m downstream of the impact region, allowing projectile reuse. This configuration enables systematic investigation of droplet impact phenomena across a range of impact velocities, projectile materials, and droplet geometries, providing the temporal and spatial resolution required for detailed analysis of collision dynamics.

### 2.2 High-speed visualization

High-speed visualization was performed using a Shimadzu HPV-X2 camera operating at 10 million frames per second with 100 ns temporal resolution, capturing 256 frames per sequence (250 × 400 pixels) in two configurations: shadowgraph view and angle view.

Shadowgraph imaging provided a spatial resolution of 16 μm/pixel using a custom 808 nm illumination source positioned behind the droplet. The illumination produced 2.5 ns pulses triggered by the camera’s exposure output signals to ensure precise temporal synchronization. In this configuration, light transmitted through the droplet underwent at least two refractions before reaching the camera, requiring correction during video data analysis. Light incident near the droplet edge was reflected away from the camera, reducing the effective field of view, such that only the central region of the droplet was observable in the shadowgraph mode. Given the refractive index of water (*n* = 1.33), image magnification effects were corrected using a factor of M = 1.33 to obtain the true physical dimensions. The imaging configuration differed for angled-view recording, where most of the droplet volume was visible, however, refractive distortions remained present and affected the apparent relative dimensions.

Angle-view video sequences were recorded at 18° and 26° from horizontal and 34 μm/pixel resolution. Front illumination was provided by a flash positioned above the camera where the exposure time is limited to the 25 ns offered by the high-speed camera.

The camera mounting system incorporated a z-stage and a two-axis translation stage for precise focusing, with illumination timing synchronized using a digital delay generator controlled by oscilloscope triggering from the projectile detection system. The flash illumination was triggered several microseconds prior to camera activation to ensure maximum brightness during imaging, thereby optimizing image quality for capturing transient phenomena.

To analyze the observed phenomena, artificial streak images were generated from the recorded image sequences. For each frame, a single pixel line (“profile”) was extracted and stacked sequentially to construct x-profile and y-profile streak images. The x-profile consists of horizontal lines passing through the droplet center, whereas the y-profile consisted of vertical lines through the projectile-droplet contact region. Because the projectile was in motion, the y-profile line position was shifted in each successive frame to track the projectile velocity. A velocity correction was further applied to compensate for optical distortions caused by refraction in this region, resulting from changes in droplet shape. After applying these corrections, several features became more clearly resolved. The resulting streak images revealed additional features that would otherwise be difficult or impossible to observe directly.

### 2.3 Impact dynamics sequence

In this study, a solid projectile impacts a stationary droplet at velocities of 80–180 m/s. Upon impact, a transient pressure field develops in both the projectile and the droplet. Inside the droplet, this pressure is partially relieved through lateral jetting (lamella spreading). At higher impact velocities, the pressure rise generates a shockwave that propagates through the droplet.

#### 2.3.1 Pressure rise and shockwave speed

The initial impact generates a water hammer pressure governed by the acoustic impedance relationship which in the first approximation is given by [48]:

(1) $$\Delta p=\rho_{0}c_{0}v_{\mathrm{imp}}$$

where $\rho_{0}$ represents the undisturbed water density (1000 kg/m^3^), $c_{0}$ denotes the sound speed in water (1481 m/s at 20 °C), and $v_{\mathrm{imp}}$ is the impact velocity. For the experimental velocity range of 80–180 m/s, this formulation predicts initial pressures between 118–266 MPa. The compression-induced increase in wavefront propagation speed requires correction through Heymann's [14] empirical relationship:

(2) $$s=c_{0}\left(1+kM_{0}\right)$$

where k ≈ 2 for water and $M_{0}=v_{\mathrm{imp}}/c_{0}$ is the impact Mach number. This correction accounts for pressure-dependent acoustic stiffening, yielding higher wavefront velocities, approximately 1641–1841 m/s across the investigated velocity range. The pressure is consequently expressed as:

(3) $$\Delta p=\rho_{0}sv_{\mathrm{imp}}$$

producing impact pressures of 131–331 MPa, values 11–24 % larger relative to classical water hammer predictions.

Eqs. (1), (2), (3) assume a planar impact configuration, whereas a real droplet impact involves a curved interface. Engel [52] proposed that the maximum pressure for a spherical drop takes the form $\Delta P=\left(\alpha_{E}/2\right)\rho_{0}sV$. With $\alpha_{E}$ near 0.4 for water the prefactor is close to 0.2, a reduction that would imply substantially lower internal wave speeds than we measure. Latter it was showed in [48] that this geometric factor arises not from the compression itself but from a subsequent displacement stage in which the liquid flows radially, and that this radial venting is what makes $\alpha_{E}/2$ < 1. At the high impact speeds in these experiments the contact line expands far faster than the compression wavefront, so the liquid is confined and compressed one dimensionally before any radial flow develops. The factor then reduces to unity and the pressure recovers the full form of Eq. (3). Further, the curved geometry in fact acts to raise the pressure rather than lower it. During early contact the contact edge expands supersonically, and the compression wavelets it generates coalesce into a shock envelope whose effective driving pressure exceeds the one dimensional prediction [42], [43]. Consequently, the shockwave velocities and pressures measured inside the droplet are expected to be even higher than the values given by Eqs. (2), (3).

#### 2.3.2 Cavitation and jetting mechanism

Upon reflection from the curved boundaries, the propagating shockwave forms caustics and typically focuses near the distal end of the droplet. At the liquid–gas (here water–air) interface, the compression wave reflects as a rarefaction wave, generating a region of large negative pressure. If sufficiently strong, this tensile pressure induces cavitation in the focal region, typically near the distal end of the droplet. The resulting bubbles cause slight outward deformation of the droplet surface which in later stages develops into a jet. The formation of a single coherent jet at the distal end requires highly idealised conditions: accurate alignment of the projectile and an impact along the droplet major axis. If any of these conditions is not satisfied, the jet may emerge misaligned relative to the major axis, fragment into a spray or be absent altogether. Cavitation itself, and the spray-like ejection that follows from the collapse of a distributed bubble cloud, are considerably less sensitive to these conditions: in the present experiments they were observed whenever the impact velocity exceeded approximately 140–150 m/s, irrespective of the droplet shape (Section 3.4).

The moving projectile continues to flatten the droplet, causing the water to spread laterally. Eventually, a thin liquid film remains on the projectile surface, often containing a central flat bubble originating from the initial air film trapped between the droplet and the projectile.

#### 2.3.3 Air entrapment

Prior to contact, the approaching projectile compresses the air between its surface and the droplet, forming a cushioning film that mediates the initial interaction. As the droplet edge makes contact with the projectile, it isolates this air layer from the surrounding gas. The trapped air contracts under the high pressure generated at the interface, collapsing into a bubble located at the center of the projectile-droplet contact region. The air cushioning dynamics and the resulting bubble volume were analyzed theoretically by Hicks and Purvis [39], who coupled a lubrication description of the gas layer with inviscid potential flow in the droplet to derive a scaling law for the entrapped bubble volume:

(4) $$V_{\mathrm{eb}}=\zeta {\left(\frac{\mu_{g}^{4}r_{d}^{5}}{\rho_{l}^{4}v_{\mathrm{imp}}^{4}}\right)}^{1/3},$$

where $\mu_{g}$ is the gas viscosity, $r_{d}$ the droplet radius, $\rho_{l}$ the liquid density, $v_{\mathrm{imp}}$ the impact velocity, and ζ=94.48 a numerical factor [39]. This expression predicts that the entrapped bubble volume $V_{\mathrm{eb}}$ scales as $r_{d}^{5/3}$ and is inversely proportional to $v_{\mathrm{imp}}^{4/3}$, confirming the observations where larger droplets trap larger bubbles while faster impacts result in reduced entrapment volume.

The model was validated against experiments at impact velocities up to approximately 15 m/s for ink-jet droplets ($r_{d}\approx 37\;\mu m$) [53] and up to 7 m/s for millimeter-sized droplets ($r_{d}\approx 2\;mm$) [34], though the authors noted that gas compressibility effects become significant at higher speeds. Liu et al. [37] characterized the contracting air film as a doughnut-shaped structure that evolves into a thick-rimmed toroidal configuration. An experimental evidence through high-speed X-ray imaging was provided in [36], demonstrating that the entrapped bubble typically contains a small daughter droplet and thus exhibits a toroidal shape, undergoing several volume oscillations before reaching equilibrium. At the low impact velocities investigated in those studies (up to approximately 10 m/s), the entrapment mechanism is well characterized; however, its behavior at high velocities such as explored in the present work (80–180 m/s), has received comparatively little attention, with the exception of [18] reporting bubble formation at the wall surface at comparable velocities.

### 2.4 Numerical model

To gain a better understanding of the complex pressure wave propagation inside the droplet, two-dimensional axisymmetric numerical simulations were performed using OpenFOAM [54].The model has already been validated in [18]. As shown in Fig. 1d, the droplet is initialized as a sphere with a radius R = 1.3 mm to simulate the impact conditions at $v_{\mathrm{imp}}$ = 165 m/s and 131 m/s. The impact process is modeled by directing the droplet against a no-slip wall. Initially, the distance between the droplet center and the wall is set to h = 1.7 mm. Both the radial width $y_{m}$ and the axial height $x_{m}$ of the computational domain are set to 9.6 mm. The computational domain is spatially discretized using a structured mesh. The mesh is progressively refined, with a grid size of Δx = 2 μm applied along the droplet trajectory and within the impact region.

The flow is governed by the compressible Navier-Stokes equations, including the conservation of mass and momentum:

(5) $$\frac{\partial \rho }{\partial t}+\nabla \cdot \left(\rho u\right)=0$$

(6) $$\frac{\partial \rho u}{\partial t}+\nabla \cdot \left(\rho uu\right)=-\nabla p+\nabla \cdot s+f_{\sigma }$$

where ρ is the fluid density, t is the time, u is the flow field velocity, and p is the field pressure, respectively. s represents the viscous stress tensor, which is given by $s=\mu (\nabla u+\nabla u^{T}-2/3\left(\nabla \cdot u\right)I)$, in which μ and I are the dynamic viscosity and identity tensors, respectively. $f_{\sigma }$ is the surface tension, which is modelled with the continuous surface force (CSF) method [55], [56].

The droplet surface is tracked using the Volume of Fluid (VOF) method. The phase fraction α is solved via the following transport equation:

(7) $$\frac{\partial \alpha }{\partial t}+\nabla \cdot \left(\alpha u\right)+\nabla \cdot \left(\alpha \left(1-\alpha \right)U_{r}\right)=\alpha \left(1-\alpha \right)\left(\frac{\psi_{g}}{\rho_{g}}-\frac{\psi_{l}}{\rho_{l}}\right)\frac{\mathrm{Dp}}{\mathrm{Dt}}+\alpha \nabla \cdot u$$

The third term on the left-hand side is the artificial compression term, which is active only in the interface region (where 0<α<1). Here, $U_{r}$ represents the relative velocity field constructed to compress the interface, counteracting numerical smearing while ensuring boundedness. The terms on the right-hand side account for the compressibility effects. Here, ρ and ψ denote the density and compressibility ($\psi_{l,g}=\partial \rho_{l,g}/\partial p$), respectively, with the subscripts l and g representing the liquid and gas phases. The Tait equation of state describes the liquid phase [57], whereas the gas phase is treated using a polytropic equation of state [58]. The source terms driven by the material derivative of the pressure, Dp/Dt, ensure that the transport of the volume fraction is consistent with the local expansion and contraction of the fluids.

## 3 Results

### 3.1 Overview of impact phenomena

The recorded video sequences consist of 256 frames, each with a spatial resolution of 250 × 400 pixels and a temporal resolution of 100 ns between successive frames. The image pixel size was approximately 16 µm in shadowgraph recordings and 34 µm for angled-view. Representative still images extracted from the videos are presented in Fig. 2, providing a general overview of the acquired data for the angled-view and the shadowgraph recordings, respectively.

**Fig. 2:**
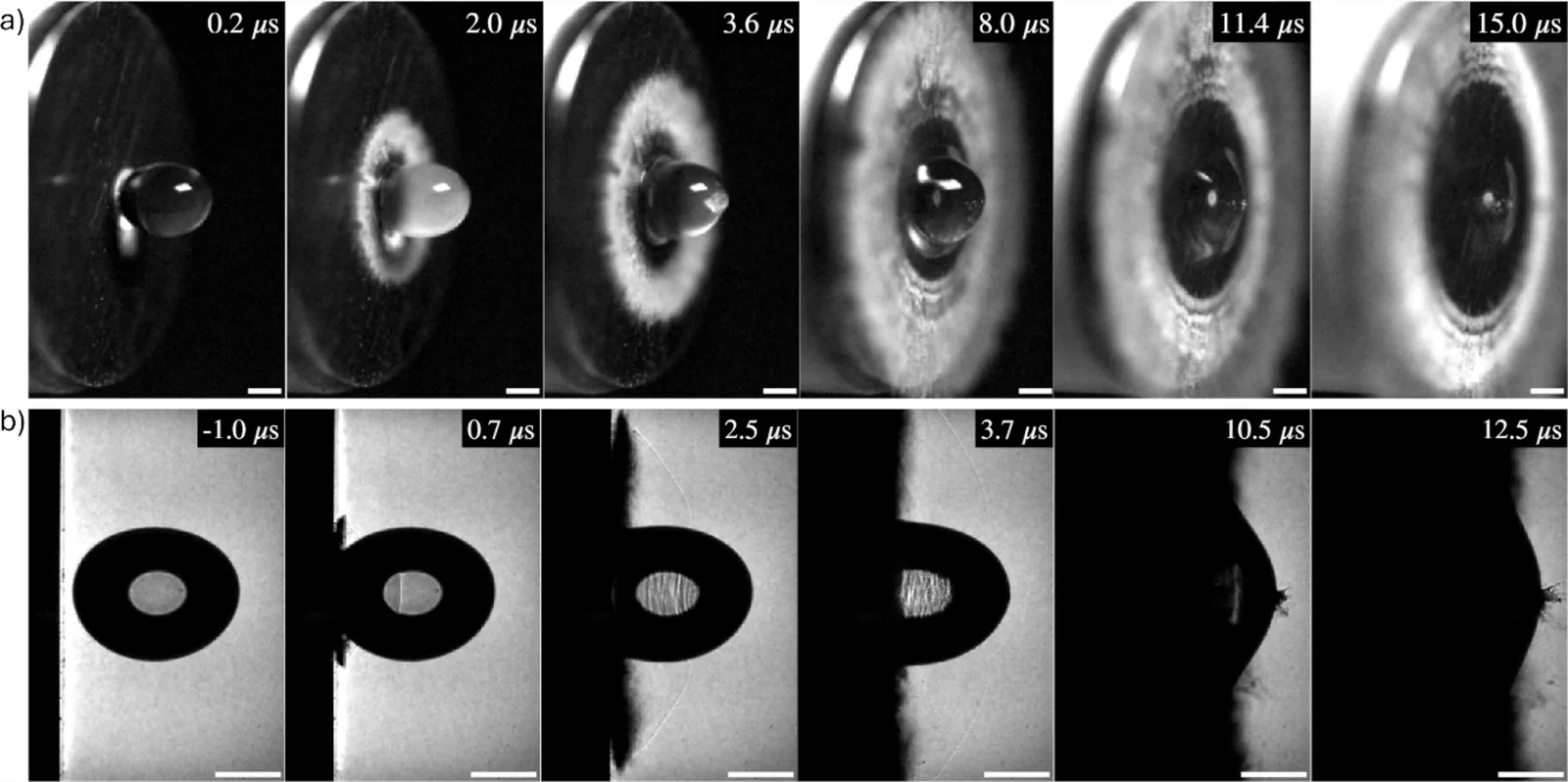
a) Representative still images for the angle-view ($v_{\mathrm{imp}}=163\;m/s,\;2r_{a}=3.2\;mm$) – see also Video 1 and b) shadowgraph experimental setup $\left(v_{\mathrm{imp}}=164\;m/s,\;2r_{a}=2.6\;mm\right)$− see also Video 2. Scale bar corresponds to 1 mm.

The images in Fig. 2a present a sequence of events for a projectile impacting a 3.2 mm droplet (axis aspect ratio 1.40) at a velocity of 163 m/s, in angle view. Immediately after impact (0.2 µs), the emergence of lateral jet (lamella) is already visible as the bright region at the impact site. As the projectile advances (2.0 µs), the lamella spreads. At this point the droplet appears cloudy owing to the white lamella shining through. The region near the droplet appears dark, indicating that the liquid there remains in contact with the projectile surface. A hydraulic-jump-like transition is observed between the dark and the white regions. At 3.6 µs, numerous bubbles are seen to have been formed at the distal end of the droplet, accompanied by a slight outward deformation (bulge) of the distal end of the droplet. By 8.0 µs, a substantial fraction of the droplet has been conveyed into the lamella. At the center of the projectile-droplet interface notably an entrapped bubble is observed, exhibiting periodic intensity fluctuations in subsequent frames. The intensity fluctuations are best observed in the video (Video 1). At 11.4 µs only a part of the initial droplet still remained at the original location. In the final image (15.0 µs) the central remnants of the droplet in the form of a thin film are travelling together with the projectile. This liquid layer is a few pixels thick (on the order of 100 µm), with the central proximal bubble remaining trapped inside the film.

The images in Fig. 2b present a comparable event, recorded in shadowgraph view. The droplet diameter $2r_{a}$ was 2.6 mm (axis aspect ratio 1.24) and the projectile velocity 164 m/s, arriving from the left as in the previous case. The first image corresponds to the moment immediately before the impact (− 1.0 µs), with impact occurring at approximately 0 µs ± 100 ns. The restriction of the field of view to the center of the droplet contrasts with the experiments by Field et al. [17] which employed cylindrical (2D) geometry and did not encounter this issue. In the frame at 0.7 µs shock wave generated upon impact is observed near the central region of the droplet, propagating to the right. Within less than 0.5 µs, another wave like feature always follows, propagating in the same direction. In subsequent frames (2.5 µs and 3.7 µs), the lamella continues spreading supersonically, generating a shockwave in air. Multiple wave reflections propagating in various directions are visible within the droplet. In general, the distally formed bubbles are not observed in the shadowgraph images, due to the curvature of the droplet preventing the observation of the droplet regions near the edge. At this point the propagating lamella already partially blocks the view. Thus, a combination of the shadowgraph and front illumination imaging is crucial for obtaining comprehensive insight into the dynamics after the impact. The presence of bubbles is nevertheless inferred even in the shadowgraph view, from the jet ejection at the droplet distal end. In this case the jet is irregular, in the form of spray. Although present, the jetting is generally less distinctly visible in the angle view sequences. At this stage, the remnants of the droplet are already traveling with the projectile, as previously described for angle view sequence (Fig. 2a).

#### 3.1.1 Streak image analysis

The streak image in Fig. 3 illustrates the temporal evolution of the horizontal central cross-section of the droplet following projectile impact, as captured in shadowgraph configuration (Fig. 2b). The image is constructed from single-pixel line profiles passing through the droplet center. The profile position is indicated by the yellow dashed line in Fig. 3a. These profiles are sequentially stacked to generate a composite representation (Fig. 3b), where the black pixels are mapped to blue and the grey levels are shown from green to red for better recognition of the features (see the colour bar in Fig. 3a). To facilitate comparison with the streak image, the frames in Fig. 3a are rotated by 90°, so that the droplet major axis is aligned with the position axis of the streak image. The result of this process is closely analogous to an experimental streak image tracking the changes along the droplet major axis in time.

**Fig. 3:**
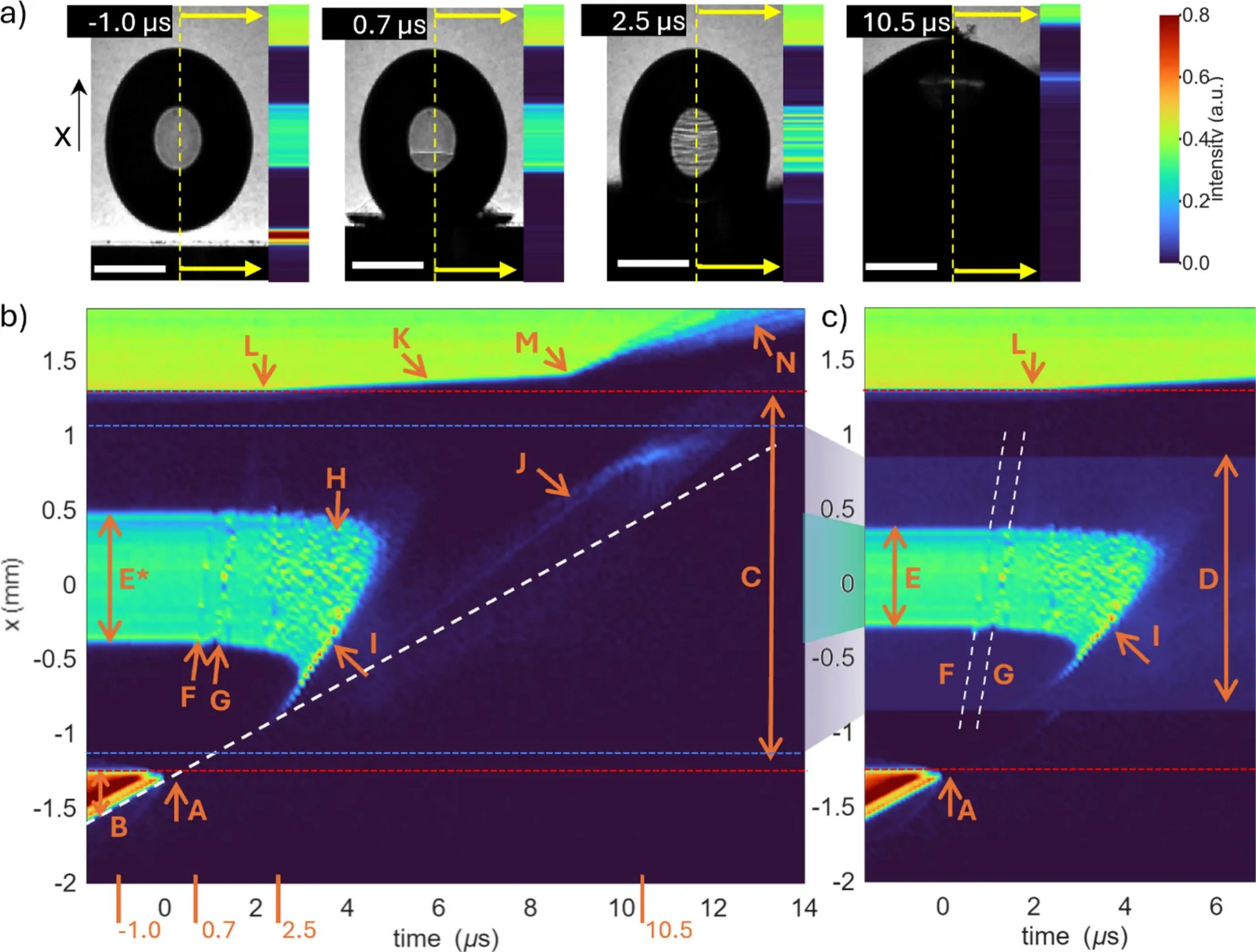
a) Four of the 256 frames, rotated by 90° so that the droplet major axis (coordinate x) is vertical and coincides with the x axis of the streak image (scale bar 1 mm). The yellow dashed lines mark the pixel lines from which the streak image is constructed. b) Artificial streak image for a projectile impact at $v_{\mathrm{imp}}=164\;m/s,\;2r_{a}=2.6\;mm$, with time on the horizontal and position on the vertical axis. The recorded grey levels are displayed in false colour, as indicated by the colour bar in (a). c) The same streak image with refraction-corrected positions, representing the real physical dimensions within the droplet. The features marked with capital letters are described in the text.

The beginning of the impact at approximately *t* = 0 is marked with (A). Prior to impact, the gap between the projectile and droplet (B) progressively decreases. The initial droplet diameter along its major axis, in direction *x* , is marked by (C). The initial positions of the extreme distal and proximal interfaces are indicated by two red dashed horizontal lines and the projectile surface trajectory the inclined white dashed line. A magnification correction accounting for optical refraction is shown in Fig. 3c, with the refraction-corrected positions, representing the real physical dimensions within the droplet, extending through (D). Owing to refractive index mismatch and focusing conditions, only the central region of the droplet is visible through the optical window (E* in the raw data streak image on the left and E in the magnification-corrected, on the right). The first (F) and second (G) wave structures propagating through the droplet are identified, marked by the two dashed white lines in the right-hand portion of the Fig. 3b to lead the eye. They are followed by subsequent reverberations arising from multiple reflections at the droplet interface (H). Additional optical distortions develop as the projectile approaches the observation window and as the lamella develops, progressively obscuring the field of view (I). A faint reflection from the lamella rim propagates together with the projectile (J).

The distal droplet interface remains stationary up to approximately 2.5 µs (L), after which it starts to expand outward slightly (K). This motion is attributed to localized deformation of the water–air interface associated with cavitation in the distal region; in many cases, this expansion is followed by local jetting. Jet ejection initiates at (M), characterized by the expulsion of liquid in a narrow filament from the distal droplet wall. When the jet exhibits an irregular morphology, as in the present case, the boundary between the jet and the surrounding air appears diffused (N). By contrast, a compact and well-defined jet produces a sharp interface.

### 3.2 Shockwaves inside the droplet

The shadowgraph streak images reveal two distinct wave structures propagating through the droplet following impact. These features can be clearly identified in the shadowgraph streak image (Fig. 3c), where the primary shockwave S1 is indicated by the white dotted line and marker (F) and the secondary wave feature S2 by marker (G). At later times, individual wavefronts can no longer be resolved, as they become obscured by acoustic reflections within the droplet (H). At lower impact velocities (below 100 m/s), the associated pressure amplitudes are expected to decrease accordingly, hindering reliable detection.

The primary shockwave S1, which originates at the projectile–droplet contact zone, is a compression front that propagates axially through the central region of the droplet. The measured velocities of S1 exhibit an approximately linear dependence on impact velocity, consistent with the empirical relation proposed by Heymann (Eq. (2). The propagation velocity increases from approximately 1500 m/s at lower impact velocities to values exceeding 2000 m/s at the highest impact velocities investigated (Fig. 4a). For the representative case of $v_{imp}=164\;m/s$, the measured velocity of S1 was 1960 m/s (Fig. 4b). A second wave feature, denoted S2, consistently emerges in the visible window approximately 500 ns after the arrival of S1 and generally exhibits a broader spatial extent (Fig. 4b). The physical nature of S2 is clarified by the simulation results presented in Fig. 4c, which show the pressure field and its gradient at two representative time instants. At t = 0.9 μs, when S1 traverses the central region of the droplet, the pressure field exhibits a well-defined compression front with a peak pressure exceeding 300 bar. At t = 1.2 μs, S1 has propagated further along the axial direction, whereas S2 appears as a distinct structure located near the droplet boundary. Importantly, the simulation results indicate that S2 is associated with negative pressure regions reaching approximately −60 bar, demonstrating that S2 corresponds to a tension wave rather than a secondary compression front.

**Fig. 4:**
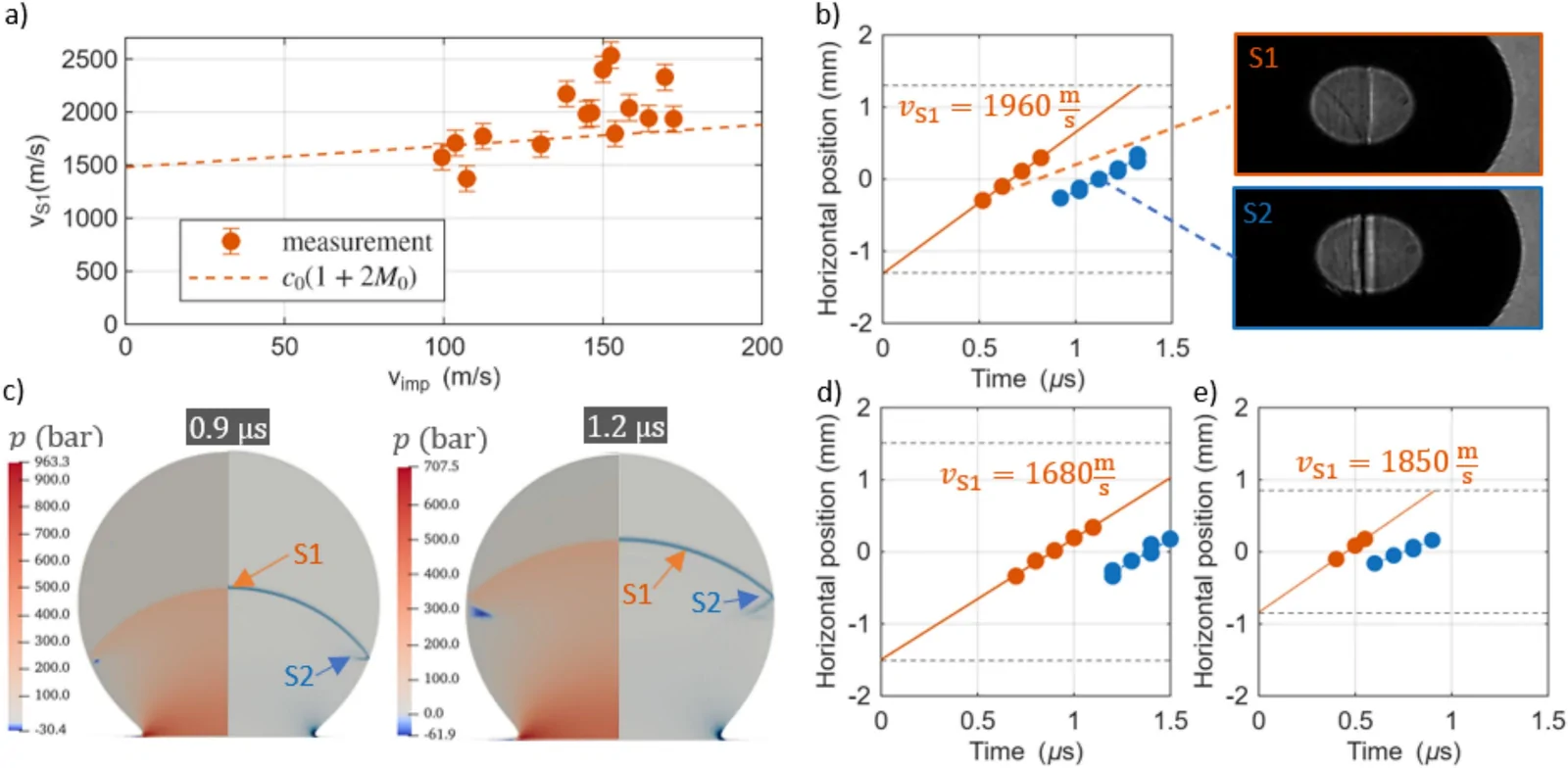
a) Dependence of shockwave velocity inside the droplet on projectile velocity$v_{imp}$ plotted together with the Eq. (2). b) Measured positions of S1 and S2 waves for a droplet impacting a steel surface at $v_{\mathrm{imp}}=164\;m/s$ and initial droplet diameter $2r_{a}=2.6\;mm$, together with representative images illustrating the transit of S1 and S2 through the droplet center. The position *x* =0 corresponds to the droplet center. c) Simulation results for $v_{\mathrm{imp}}=165\;m/s$, showing the pressure field (left half) and its gradient (right half) at t = 0.9 μs and t = 1.2 μs, corresponding to the transit of S1 and S2 through the droplet center. d) Same as in b), for $v_{\mathrm{imp}}=131\;m/s,\;2r_{a}=2.9\;mm$. e) Same as in b), for $v_{\mathrm{imp}}=172\;m/s,\;2r_{a}=1.7\;mm$.

This tensile wave originates from the interaction between the primary compression front S1 and the curved free surface of the droplet. As S1 propagates outward from the impact zone, it encounters the water–air interface at oblique incidence angles along the droplet periphery. At this free boundary, the compression wave is partially reflected as a rarefaction wave, generating a region of tensile stress that propagates along the inner droplet surface. Since the reflected tension wave travels near the curved boundary rather than through the bulk interior, its geometric path is greater, leading to a delayed appearance in the shadowgraph images relative to S1. In interpreting both experimental and simulation results, it should be noted that the geometry combines cylindrical and spherical characteristics: the droplet itself is spherical, whereas the numerical simulations represent a two-dimensional central cross section of the droplet. Consequently, the S2 wave feature observed near the boundary in the simulation corresponds to a wavefront that is in fact distributed azimuthally around the spherical droplet. The position-time trajectories of S1 in the representative cases shown in Fig. 4(b, d, e) yield $v_{S1}=1680\;m/s$ at $v_{\mathrm{imp}}=131\;m/s$ (Fig. 4d), $v_{S1}=1960\;m/s$ at $v_{\mathrm{imp}}=164\;m/s$ (Fig. 4b) and $v_{S1}=1850\;m/s$ at $v_{\mathrm{imp}}=172\;m/s$ (Fig. 4e). The velocities are obtained from linear fits to wavefront positions determined to within about one pixel (16 µm) at a frame interval of 100 ns, which corresponds to an uncertainty of about 10 % (approximately 160 m/s). Individual cases should therefore be regarded as examples, while the dependence on impact velocity is represented by the full data set in Fig. 4(a), which shows an overall increase of $v_{S1}$ with $v_{\mathrm{imp}}$.

This velocity dependence reflects the nonlinear equation of state of compressed water, consistent with the Tait formulation, in which the speed of sound increases with pressure [59].

The identification of S2 as a surface-guided tension wave has direct implications for the subsequent cavitation dynamics described in Section 3.3. The tensile stresses generated by this reflected rarefaction wave, particularly in regions where geometric focusing along the curved interface leads to convergence toward the distal end of the droplet and promotes the nucleation of cavitation bubbles in that region. The temporal sequence, in which the primary compression wave S1 reaches the distal end first, followed by the converging tension part of the wave S2, is consistent with the experimentally observed incubation interval between shockwave transit and the onset of cavitation at the distal end.

### 3.3 Cavitation bubbles at the distal end

A direct consequence of shockwave propagation, reflection, phase reversal, and interference within the droplet is the formation of regions of negative pressure, most pronounced near the distal droplet interface. When the tensile stress exceeds a critical threshold, cavitation bubbles are nucleated. The mechanism governing cavitation at the distal end is rooted in the reflection behavior of acoustic waves at the droplet boundary [31], [32]. As the compression shock wave propagates approximately spherically from the impact site toward the distal end, it progressively interacts with the curved water–air interface. Owing to the large acoustic impedance mismatch between water and air, the incident compression wave is reflected as an expansion (rarefaction) wave (S2 on Fig. 4b). Importantly, this reflection process occurs continuously along the droplet boundary and not exclusively at the distal apex. Consequently, expansion waves are generated well before the primary shock reaches the far end of the droplet [23].

Although expansion waves are generated almost immediately once the compression front interacts with the droplet boundary, cavitation bubbles are observed exclusively in the distal region rather than throughout the droplet interior. This spatial selectivity arises from the geometric focusing characteristics of the curved interface. Near the proximal region, the reflected expansion waves diverge or propagate without significant convergence, resulting in tensile stresses that remain below the cavitation threshold. As the compression front advances toward the distal end, it reflects from progressively more distal segments of the boundary. The curved distal interface effectively acts as a concave acoustic mirror, focusing the reflected rarefaction waves into a caustic region near the distal wall. This caustic focusing mechanism is analogous to the acoustic wave concentration observed in laser-induced cavitation experiments involving curved interfaces, where concave geometries similarly concentrate rarefaction waves toward localized interior regions, leading to cavitation at well-defined spatial locations [60], [61], [62].

Within this focal region, the superposition of converging rarefaction waves amplifies the local tensile stress to values that may exceed the critical threshold for heterogeneous cavitation nucleation. For water containing dissolved gases and particulate impurities, this threshold ranges from approximately −1 to −30 MPa, depending on the size and nature of pre-existing nuclei [63]. The lower bound corresponds to micron-scale gas inclusions, whereas the upper bound is characteristic of carefully purified water containing only nanoscale nucleation sites [64]. The cavitation bubbles therefore nucleate preferentially where geometric focusing maximizes the negative pressure amplitude, which occurs near the distal end of the droplet. Notably, the focusing process may generate multiple successive tensile phases rather than a single rarefaction pulse. It was demonstrated in laser-induced cavitation experiments near a curved water surface [60] that reflected waves produce distinct tensile phases at different spatial locations and times, with later phases capable of nucleating additional cavitation events, even after the initial bubble population already formed. Such multi-phase focusing behavior is consistent with the experimentally observed formation of spatially distributed bubble clouds, rather than isolated cavitation sites, at the distal end of impacting droplets (Fig. 5).

**Fig. 5:**
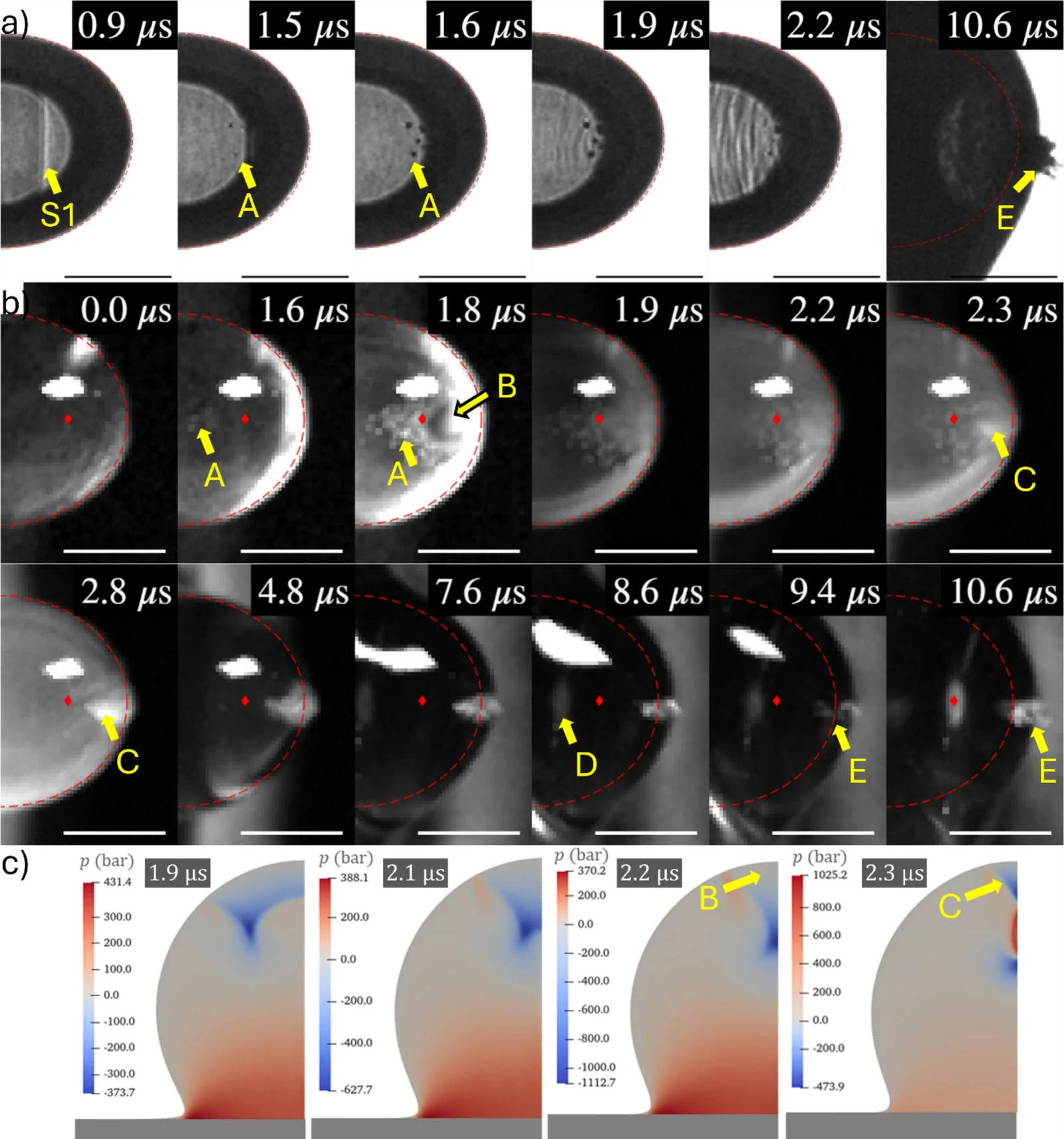
Cavitation bubble formation at the distal region of the droplet. a) Shadowgraph visualization for $v_{\mathrm{imp}}=173\;m/s,\;2r_{a}=2.49\;mm$. b) Angle-view recording (18°) for $v_{\mathrm{imp}}=167\;m/s,\;2r_{a}=2.6\;mm$. The red dashed line indicates the initial droplet position, and the red diamond marks the right focal position of the fitted ellipse. The scale bar corresponds to 1 mm. c) Comparison with the modeled pressure distribution for $v_{\mathrm{imp}}=165\;m/s,\;2r_{a}=2.6\;mm$. Yellow arrows labeled A indicate cavitation bubbles, B denotes the high-pressure region corresponding to the dark spot within the cavitation cloud at the distal end, C marks the region of tightly clustered cavitation bubbles, D indicates a trapped air bubble at the impact surface and E denotes the spraying jet.

The bubbles are directly observed in the angle-view recordings (Fig. 5), and they also generate a characteristic signature in the corresponding streak images (Fig. 3). In streak images derived from shadowgraph recordings, a slight outward bulging of the droplet wall becomes visible (Fig. 3b, marker K) following an incubation interval after impact, which can be attributed to bubble nucleation and growth. The temporal sequence of events supports a causal link between shockwave propagation and cavitation formation. About 1.2–1.5 µs after impact (depending on droplet size), the primary shockwave is reflected at the distal free surface as a tensile wave, while the S2 tension wave converges on this region along the droplet boundary. Owing to the distal curvature, the two partially overlap and arrive at different locations at slightly different times, so bubble nucleation and growth result from their combined action, depending on droplet size and sphericity.This timing is consistent with numerical predictions of pressure field evolution during droplet impact [18], [23].

Fig. 5a illustrates cavitation bubble formation near the distal end of a 2.5 mm droplet ($v_{\mathrm{imp}}=173\;m/s$) using shadowgraph imaging. At 0.9 µs after impact, a primary shockwave S1 can be observed propagating toward the distal end. At 1.5 µs, the first cavitation bubbles become visible. By 1.9 µs, these bubbles are encountered by the reflected primary shockwave returning from the distal boundary, which scatters upon interaction with the cavitation bubble cloud.

Fig. 5b presents a sequence of images showing the dynamics near the distal region of a 2.6 mm droplet ($v_{\mathrm{imp}}=167\;m/s$) recorded at an observation angle of 18°. Cavitation bubbles first appear 1.6 µs after impact and subsequently expand to form a conically shaped cavitation bubble cloud oriented away from the distal surface. At 2.3 µs, a brighter region emerges within the cavitation cloud, corresponding to the coalescence of individual bubbles into a more compact structure. This coalesced cavity continues to grow until approximately 2.8 µs, while the optical intensity associated with the primary cavitation cloud gradually decreases. Between 2.8 µs and 9.4 µs, a collapse motion directed toward the distal end of the droplet is observed, ultimately leading to jet formation at *t* = 10.6 µs (Fig. 5b). A direct comparison of the cavitation cloud shapes in Fig. 5(b) with the computed pressure field in Fig. 5(c) is limited by the viewing angle (18°) and light refraction at the curved droplet surface, which shift the apparent positions of features, by the ellipsoidal shape of the experimental droplets (the simulation assumes a sphere), and by the images being projections of the whole droplet volume rather than a two-dimensional central cross-section. Nevertheless, experiment and simulation consistently show that cavitation onset coincides with the tensile loading of the distal region by the reflection of S1 at the distal free surface and by the S2 tension wave converging along the droplet boundary. Owing to the distal curvature, these contributions reach different parts of the region at different times and partially overlap. Cavitation onset and cloud evolution thus result from their combined action, with the exact sequence depending on droplet size and sphericity.

The transition from individual bubbles to a prolate cavity elongated along the wave propagation axis is consistent with the cavity dynamics reported in [60], where it was observed that laser-induced cavitation near curved surfaces produces elongated cavities aligned with the acoustic focusing direction, which subsequently collapse asymmetrically to generate directed liquid jets.

### 3.4 Jet formation

The propagating shockwaves may repeatedly revisit the bubble-populated region, after successive boundary reflections. When they manifest as rarefaction waves, they can promote further expansion of pre-existing bubbles, and compression waves may initiate or enhance the jet formation at the distal droplet interface. The results from droplet impacts with the stainless-steel surface indicate the existence of a distinct impact velocity threshold (140–150 m/s), below which no jetting is detected and above which jet formation occurs reliably. This threshold is likely a combination for cavitation nucleation and forced non-spherical collapse near the free interface. The jetting mechanism may involve a process analogous to the “water bell” structure described in [60], in which the collapse of an elongated cavity near a curved boundary produces an annular liquid sheet that converges into a focused central jet.

Three cases are analyzed comparatively in Fig. 6: one below the threshold (no jetting) and two above threshold. Because the three cases differ in both impact velocity and droplet size, the position of the most distal droplet point in Fig. 6(a3)–(c3) is shown in normalised form, $x{/(2r}_{a})$, as a function of the normalised time ${t/(2t}_{c})$, where $t_{c}=r_{a}/v_{\mathrm{imp}}$ is the time in which the projectile travels a distance equal to the droplet major semi-axis $r_{a}$. At ${t/(2t}_{c})=1$ the projectile has thus travelled a distance equal to the initial droplet length $2r_{a}$. In this representation the onset of the distal bulge, the time $t_{s1}$ at which the shock wave reaches the distal end, and the subsequent motion of the distal interface can be compared directly between the three cases. Jets consistently emerge from the distal end of the droplet and are invariably preceded by the characteristic outward deformation (bulging) of the distal interface. For relatively small droplets (< 2 mm, Fig. 6(c1)-(c4)), the resulting jet is typically slender and exhibits sharply defined boundaries. A second jet (J2) frequently emerges along the axis of the primary jet, characterized by a smaller diameter but comparable velocity. in some cases, a third jet (J3) is also observed (see, e.g., Fig. 6(c2). The process may culminate in multidirectional spraying, occurring 7–8 µs after impact (example shown in Fig. 6(b2). For medium-sized and large droplets (> 2 mm, second row in Fig. 6(a1)-(a4) and (b1)-(b4), distal initial deformation is likewise observed. However, the subsequent ejection morphology differs markedly. In most cases, the formation of thin, well-defined jets is largely absent. Instead, after an incubation period, liquid is expelled predominantly in the form of a spray. This spray emission occurs with a highly reproducible delay of 8 µs ± 0.5 µs, closely matching the spray onset timing observed in smaller droplets.

**Fig. 6:**
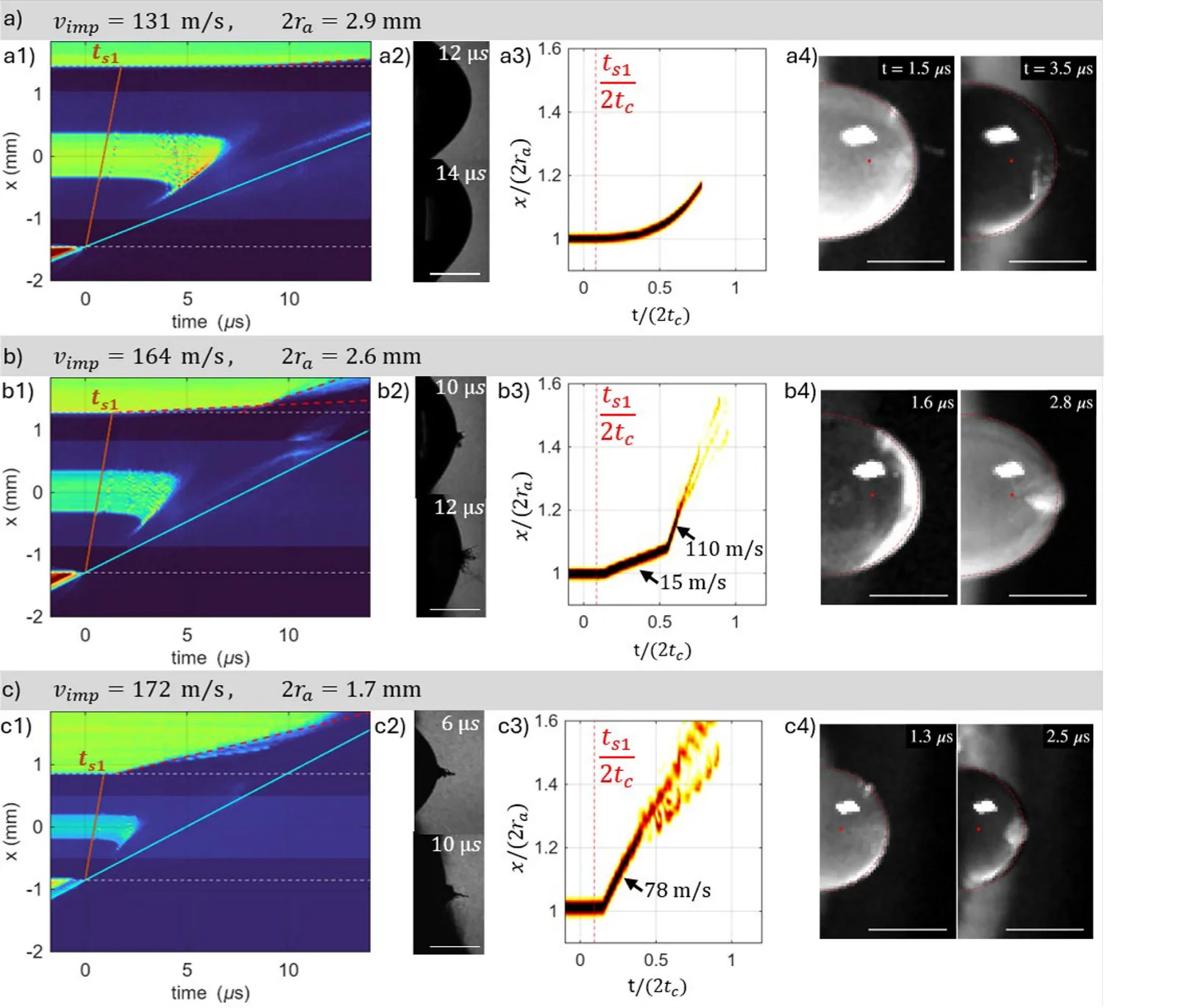
Artificial streak images (left column) with corresponding selected shadowgraph images captured during the late/jetting phase (second column), temporal evolution of the droplet most distal point (normalized to droplet diameter $2r_{a})$ in normalized time ${t/(2t}_{c})$ with $t_{c}=r_{a}/v_{\mathrm{imp}}$ (third column) and selected angle-view images for comparable events (fourth column). The $t_{s1}$ is estimated time in which the shockwave reaches the distal end of the droplet. Cases: a) $v_{\mathrm{imp}}=131\;m/s,\;2r_{a}=2.9\;mm$, b) $v_{\mathrm{imp}}=164\;m/s,\;2r_{a}=2.6\;mm$, and c) $v_{\mathrm{imp}}=172\;m/s,\;2r_{a}=1.7\;mm$ for which $t_{s1}$ are 1.8 μs, 1,3 μs and 0,9 μs respectively. Corresponding shadowgraph images are shown in a2), b2), and c2), respectively. The position of the most distal droplet point is plotted in a3), b3), and c3) for each case. Comparable events recorded in the angle-view configuration are presented in a4) $v_{imp}=146\;m/s,\;2r_{a}=2.8\;mm$, b4) $v_{imp}=167\;m/s,\;2r_{a}=2.6\;mm$ and c4) $v_{\mathrm{imp}}=163\;m/s,\;2r_{a}=2.0\;mm$. Scale bars correspond to 1 mm.

The observed dependence of jet morphology on both impact velocity and droplet size can be understood through the interplay between shock wave focusing intensity and droplet geometry. Below the velocity threshold of approximately 140–150 m/s, the compression wavefront does not attain sufficient amplitude to generate the tensile stresses required for cavitation nucleation at the distal end. Consequently, neither bubble formation nor jetting is observed. Above this threshold shock waves of sufficient strength are generated to induce cavitation through reflection and focusing at the distal boundary.

The distinction between coherent jet formation in smaller droplets (<2 mm) and spray-like ejection in larger droplets (>2 mm) reflects differences in the quality of geometric focusing. Smaller droplets, which are typically nearly spherical (ellipticity < 1.1), produce a more confined acoustic focal region near the distal interface. The resulting compact cavitation zone undergoes asymmetric collapse, producing a well-defined, high-velocity jet. In contrast, larger droplets generally exhibit greater ellipticity (typically > 1.1, attributable to acoustic levitation forces), which introduces aberrations in the wave focusing geometry. The more elongated droplet shape distributes the reflected wave energy over a broader focal volume, leading to the formation of spatially dispersed cavitation cloud rather than a localized bubble cluster. The subsequent collapse of this distributed bubble population drives liquid ejection from multiple sites simultaneously, giving rise to the experimentally observed spray-like morphology.

Considering that the outward deformation leading to the jet is caused by the growth of bubbles very close to the interface we can estimate an averaged pressure in this region. The deformation velocities in Fig. 6(b3) and (c3) are approximately constant thus we can ignore the acceleration. Let us consider that the bulge is formed by a bubble expanding in close proximity due to a constant negative pressure and ignore any non-sphericity. Then we can equate pressure needed to drive the bubble expansion from the Rayleigh equation as:

(8) $$\Delta P=\frac{3}{2}\rho \dot{R^{2}}$$

where $\dot{R}$ is the bubble’s radial velocity and $\Delta P=p_{B}-p_{\infty }$ the pressure difference between bubble wall and far away from it. Substituting the local radial velocity for the measured velocity in case Fig. 6 (b3) of 15 m/s we obtain a pressure difference of ΔP = 3.4 bar and for 78 m/s in Fig. 6(c3) ΔP  = 91 bar.

The temporal sequence of events leading to jet formation is summarized in Fig. 7. The timing of each phenomenon is normalized to the characteristic impact timescale $t_{c}=r_{a}/v_{\mathrm{imp}}$ where $t/2t_{c}=1$ corresponds to the projectile traveling a distance equal to the droplet major axis diameter $2r_{a}$. For small droplets, the normalized occurrence times of shockwave detection, distal bulge onset, and jet initiation are well-defined. The first jet (J1) typically appears at approximately $t/2t_{c}\approx 0.25$, followed by secondary jets (J2 and, occasionally, J3) at later normalized times, with spray formation occurring as the final stage. For medium and large droplets, the normalized temporal evolution exhibits a similar overall ordering from shockwave propagation to bulge development and subsequent liquid ejection. However, for medium and large droplets, coherent individual jets are generally absent, and the dominant ejection mode is spray emission. The reproducible normalized timing of spray onset across droplet size categories indicates that the governing wave propagation dynamics scale with the characteristic timescale $t_{c}$, whereas the transition between jet and spray morphologies is controlled primarily by geometric focusing quality, as discussed above.

**Fig. 7:**
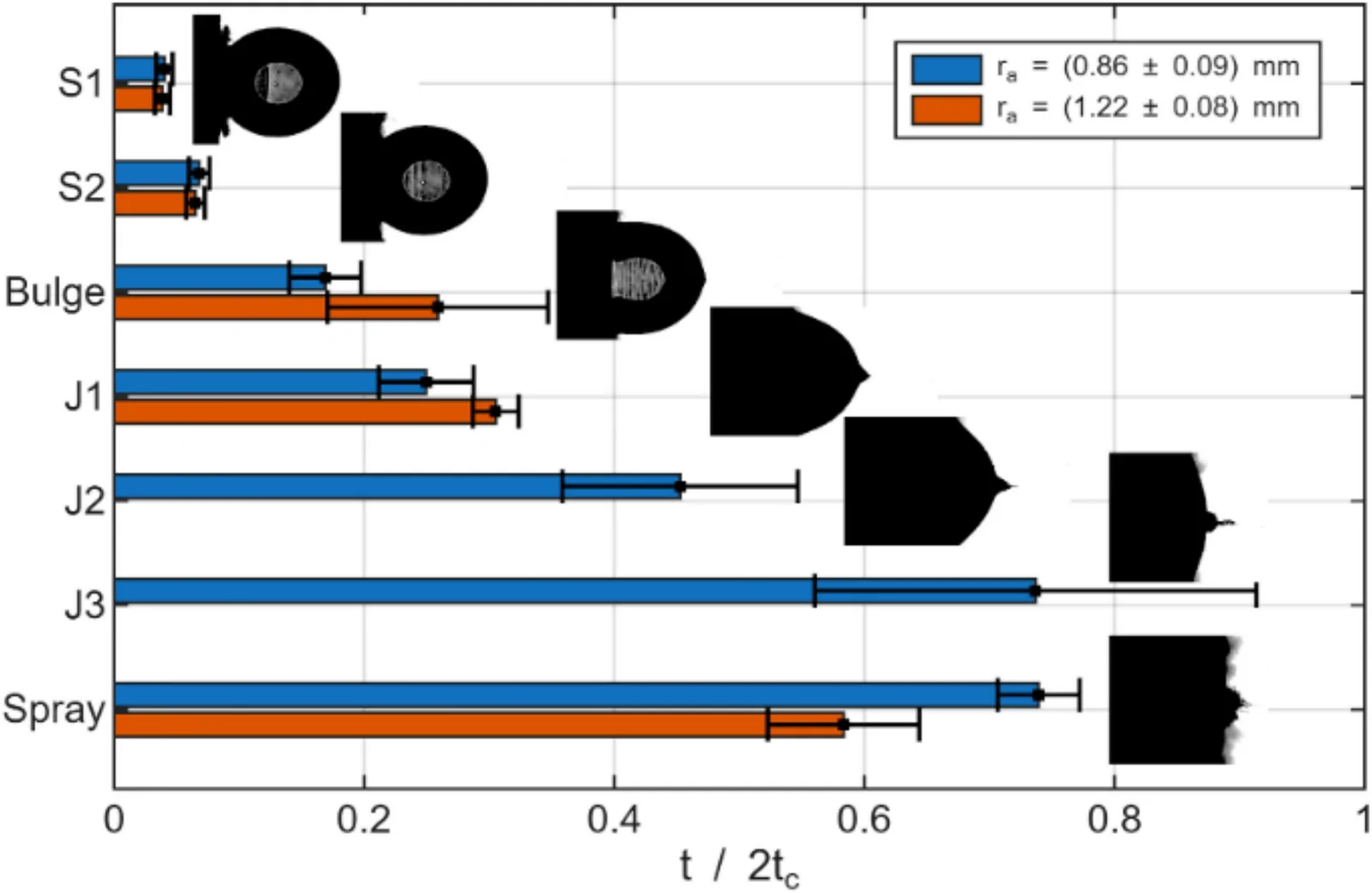
Normalized timing ($t_{c}=r_{a}/v_{\mathrm{imp}}$) for shockwave detection (S1 and S2), onset of bulging and of different jetting regimes. Blue: small droplets with $r_{a}=0.86\;mm\;\pm \;0.09\;mm$ and $t_{c}=5.2\;\mu s\;\pm \;0.5\;\mu s$. Red: large droplets with $r_{a}=1.22\;mm\;\pm \;0.08\;mm$ and $t_{c}=7.6\;\mu s\;\pm \;0.6\;\mu s$.

### 3.5 Entrapped bubble at the proximal end

As noted previously, certain impact-related phenomena are more clearly resolved in a video as compared to sequences of individual frames. This becomes evident for the air entrapment between the projectile and the droplet. During impact, the thin layer of air is compressed ahead of the approaching projectile and becomes isolated from the surrounding air as the droplet contacts the projectile surface along the periphery. This trapped air subsequently contracts into a bubble located near the center of the projectile–droplet interface.

The bubble is visible in angled-view recordings a few microseconds after the impact. Consistent with previous studies [36], it typically contains a small daughter droplet and therefore exhibits a toroidal morphology. Although the structure is relatively small (approximately 100 µm in characteristic size laterally), the experimental spatial resolution of 16 µm/px allows its temporal evolution to be resolved.

Analysis of the video sequence (Video 1) shows that the bubble intensity exhibits periodic fluctuations in time. This behavior is consistent with the findings of Lee et al. [36], where the bubble undergoes several shape oscillations before reaching equilibrium. These oscillations modulate the amount of light reflected from the bubble interface, producing the flickering intensity pattern observed in the video recordings. The overall bubble brightness was quantified by summing the pixel intensity values within a 25 × 20 px region encompassing the bubble. The resulting intensity variations in time are plotted for three representative cases shown in Fig. 8: (A) $v_{\mathrm{imp}}=163\;m/s,\;2r_{a}=3.2\;mm$, (B) $v_{\mathrm{imp}}=163\;m/s,\;2r_{a}=2.0\;mm$ and (C) $v_{\mathrm{imp}}=146\;m/s,\;2r_{a}=2.8\;mm$. Case (A), shown in Fig. 8a by the blue curve, corresponds to the event depicted in Fig. 2a. Representative images of the bubble at high-intensity and low-intensity state for this event are presented in Fig. 8b and Fig. 8c, respectively.

**Fig. 8:**
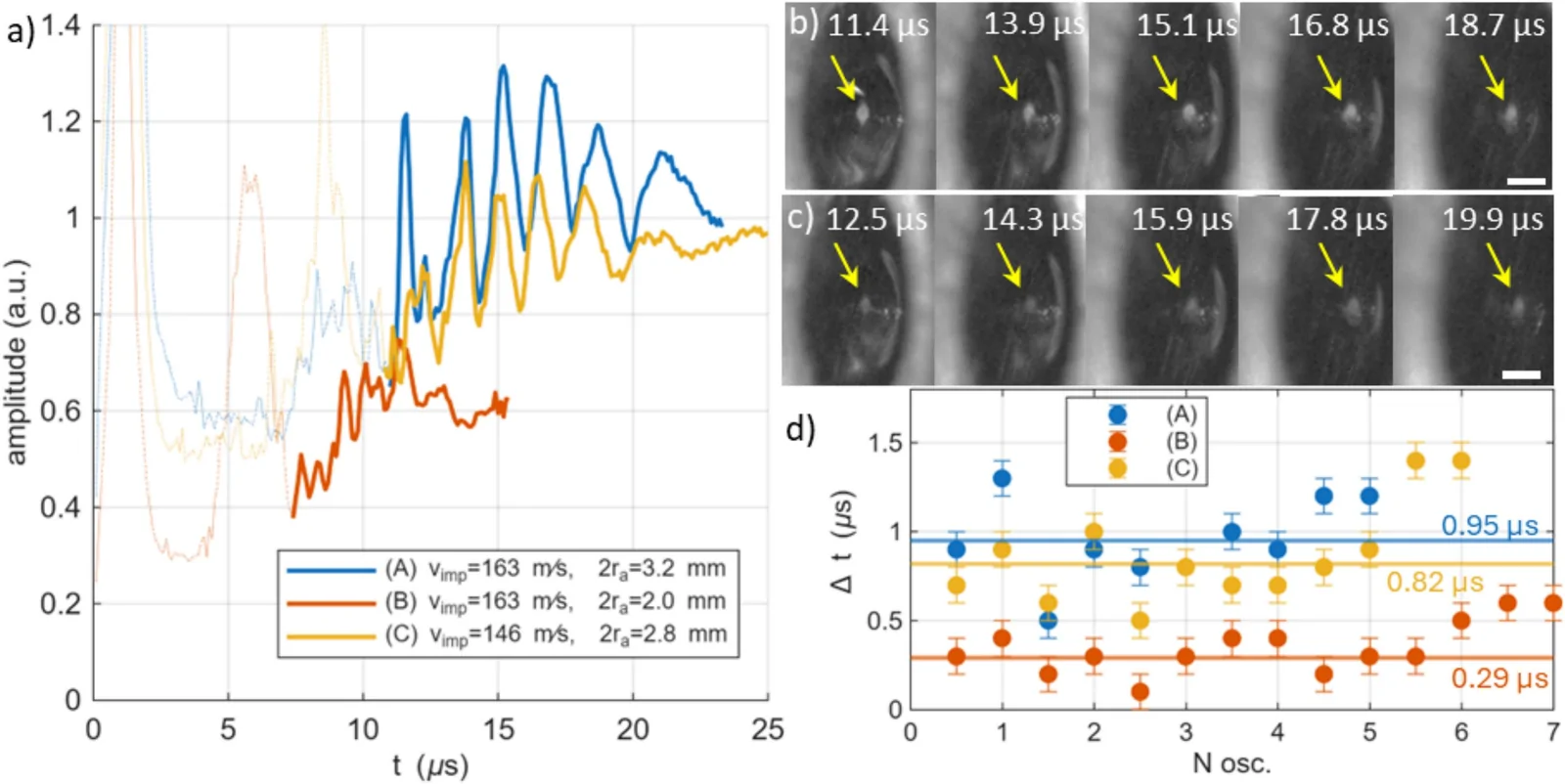
a) Cumulative intensity calculated within a 25 × 20 pixel region representing the entrapped bubble ring at the droplet–surface boundary as a function of time after impact. Representative images for case (A) are shown for b) high-intensity and c) low-intensity ring states. The scale bar corresponds to 1 mm. d) Time interval between successive intensity extrema as a function of oscillation number (N osc.), for all three cases, together with averaged half period of the observed flickering.

Fig. 8a shows the continuous intensity signal, from which the occurrence times of the intensity minima and maxima were extracted for times after 8 μs to avoid contributions from lamella formation and flash reflections. Their relative onset is plotted in Fig. 8d, from which the bubble oscillation frequency was determined. For the three cases shown in Fig. 8, the mean oscillation frequencies were 0.53 MHz for case (A), 1.72 MHz for case (B) and 0.61 MHz for case (C). In all three cases the decrease in oscillation frequency was observed toward the later stage of the impact process. The characteristic impact times $t_{c}$ were 9.8 μs, 6.2 μs and 9.6 μs for cases (A), (B) and (C), respectively. The three cases span different combinations of impact velocity and droplet diameter. Using Eq. (4), the predicted entrapped air volumes for cases (A) and (C) are comparable ($V_{C}/V_{A}\approx 0.93$) while case (B) yields a distinctly smaller value ($V_{B}/V_{A}\approx 0.46$). This grouping is consistent with the measured oscillation frequencies: cases (A) and (C) oscillate at comparable frequencies of 0.53 and 0.61 MHz, respectively, while case (B) oscillates at the distinctly higher frequency of 1.72 MHz, as expected for a smaller entrapped bubble. The correspondence indicates that the parametric dependence on droplet radius and impact velocity in Eq. (4) captures the scaling trends even at velocities well beyond the original validation range of the model. The predicted absolute volumes, however, correspond to equivalent spherical radii of approximately 2 to 3 µm, which are physically inconsistent with the observations. The bubble structure is visually resolved with a lateral extent of approximately 100 µm. This discrepancy suggests that the assumptions underlying Eq. (4), including incompressible lubrication flow in the gas layer, may break down in this regime. In particular, gas compressibility, surface tension effects, and deviations from the viscous–inviscid asymptotic limit are all expected to contribute to the observed differences.

## 4 Conclusions

This study reports direct experimental observations of shockwave propagation, cavitation bubble formation, jet generation and entrapped bubble oscillation inside liquid water droplets during high-speed impact on solid surfaces at velocities between 80 m/s and 180 m/s.

Two distinct wave structures were resolved in the early stage within the droplet interior using shadowgraph images and corresponding streak image analysis. The primary structure S1 is a compression front originating at the projectile–droplet contact, with measured propagation velocities ranging from approximately 1500 m/s to values exceeding 2000 m/s. These velocities show an overall increase with impact velocity, consistent with pressure-dependent acoustic speed correction. The secondary wave structure S2 consistently appears approximately 500 ns after the primary wave and was identified through complementary numerical simulations as a tension wave generated by the reflection of the compression front at the curved water–air interface. Because the reflected rarefaction propagates along the droplet periphery rather than through the central bulk, it follows a longer geometric path and therefore arrives with a characteristic time delay.

The curved distal droplet wall functions as a concave acoustic reflector, focusing the converging rarefaction waves and forming a caustic region near the distal wall. When the resulting tensile stress exceeds the heterogeneous nucleation threshold, cavitation bubbles form in this focal zone. Cavitation onset occurs approximately 1.2 to 1.5 µs after impact, when the primary shockwave S1 reflected at the distal surface and the converging S2 tension wave partially overlap. Since their relative timing varies along the curved distal surface and with droplet size and sphericity, onset results from their combined, locally varying action. The experimentally observed formation of spatially distributed bubble clouds, rather than single cavitation sites, is consistent with focusing behavior involving multiple temporal phases, in which distinct tensile phases develop at different spatial locations and times.

The observations indicate that the asymmetric collapse of cavitation structures, generated by the passage of the shock wave, drives the directed liquid jet from the distal droplet region. Jet morphology exhibits distinct dependence on droplet geometry: smaller, nearly spherical droplets (<2 mm, ellipticity <1.1) produce well defined jets, with secondary jets (J2, J3) frequently observed. In contrast, larger, more elongated droplets (> 2 mm, ellipticity > 1.1) distribute the reflected waves over a broader focal volume, leading to formation of dispersed cavitation clouds. The collapse of this distributed bubble population results in spray-like liquid ejection from multiple sites simultaneously.

Entrapped air bubble at the proximal projectile–droplet wall was characterized through light-intensity fluctuation measurements. The entrapped bubble exhibits periodic oscillations with frequencies ranging from 0.5 MHz to 1.8 MHz. Impact cases where the combination of impact velocity and droplet radius returned similar predicted entrapped volumes also exhibited similar oscillation frequencies, while a case with a distinctly smaller predicted volume oscillated at a higher frequency.

The combined use of shadowgraph and angle-view ultra-high-speed imaging at 10 million frames per second was essential for obtaining a comprehensive characterization of the internal droplet dynamics. Shadowgraph recordings resolve the sequence of shockwave propagation and reflection, whereas angle-view recordings capture the cavitation bubble evolution and distal jet formation. Together with artificial streak image analysis these complementary diagnostics provide a detailed experimental characterization and enable reconstruction of the impact process, from the initial pressure rise and shockwave generation through acoustic focusing and cavitation nucleation to final jet ejection. The resulting data can serve as benchmark experimental evidence for validating numerical models of compressible droplet impact. The present experiments do not establish a link between the observed internal phenomena and erosion damage. They show, however, that cavitation does occur inside droplets impacting at these velocities, which points to a possible additional source of localized loading [29]; its relevance for the erosion of wind turbine blades, steam turbine components and aerospace structures remains to be assessed.

## CRediT authorship contribution statement

**Vid Agrež:** Writing – review & editing, Writing – original draft, Visualization, Validation, Methodology, Investigation, Formal analysis, Data curation, Conceptualization. **Silvestre Roberto Gonzalez-Avila:** Writing – review & editing, Writing – original draft, Validation, Methodology, Investigation, Conceptualization. **Darja Horvat:** Writing – review & editing, Writing – original draft, Visualization, Validation, Methodology, Investigation, Formal analysis, Data curation. **Run-Ze Xie:** Writing – review & editing, Writing – original draft, Visualization, Validation, Methodology, Investigation. **Qingyun Zeng:** Writing – review & editing, Writing – original draft, Visualization, Validation, Methodology, Investigation, Formal analysis, Conceptualization. **Rok Petkovšek:** Writing – review & editing, Writing – original draft, Visualization, Validation, Supervision, Resources, Project administration, Methodology, Investigation, Funding acquisition, Formal analysis, Data curation, Conceptualization. **Claus-Dieter Ohl:** Writing – review & editing, Writing – original draft, Visualization, Validation, Supervision, Resources, Project administration, Methodology, Investigation, Funding acquisition, Formal analysis, Data curation, Conceptualization.

## Declaration of competing interest

The authors declare that they have no known competing financial interests or personal relationships that could have appeared to influence the work reported in this paper.

## Appendix A Supplementary data

Supplementary video 1 Angle-view recording of projectile at vimp=163 m/s hitting the droplet with the major axis diameter of 2r_a_=3.2 mm.

Supplementary video 2 Shadowgraph recording of projectile at vimp=164 m/s hitting the droplet with the major axis diameter 2r_a_=2.6 mm.

## References

[1] Hao H., Domenech L., Sánchez F. (2026). Modelling rain erosion surface damage initiation in turbine blades based on inspection data at wind farms. Results Eng..

[2] Worthington A.M. (1876). On the forms assumed by drops of liquids falling vertically on a horizontal plate. Proc. R. Soc. Lond..

[3] Lesser M.B., Field J.E. (1983). The impact of compressible liquids. Annu. Rev. Fluid Mech..

[4] Rein M. (1993). Phenomena of liquid drop impact on solid and liquid surfaces. Fluid Dyn. Res..

[5] Lesser M. (1995). Thirty years of liquid impact research: a tutorial review. Wear.

[6] Yarin A.L. (2006). Drop impact dynamics: splashing, spreading, receding, bouncing…. Annu. Rev. Fluid Mech..

[7] Josserand C., Thoroddsen S.T. (2016). Drop impact on a solid surface. Annu. Rev. Fluid Mech..

[8] Kyriazis N., Koukouvinis P., Gavaises M. (2018). Modelling cavitation during drop impact on solid surfaces. Adv. Colloid Interface Sci..

[9] Marzbali M., Yeganehdoust F., Ibrahim M.E., Tarasi F., Jadidi M. (2023). Liquid–solid impact mechanism, liquid impingement erosion, and erosion-resistant surface engineering: a review. Coatings.

[10] Cheng X., Sun T.-P., Gordillo L. (2022). Drop impact dynamics: impact force and stress distributions. Annu. Rev. Fluid Mech..

[11] Mohammad Karim A. (2023). Physics of droplet impact on various substrates and its current advancements in interfacial science: a review. J. Appl. Phy..

[12] Fujisawa N. (2025). Review: fundamentals of liquid droplet impingement and rain erosion of wind turbine blade. Next Energy.

[13] Vozhakov I.S., Misyura S.Y., Morozov V.S., Piskunov M.V., Piskunova A.E., Orlova E.G., Mullyadzhanov R.I. (2025). High-speed droplet impact on solid surfaces: review on the prospects of modeling fluid flow. Exp. Fluids.

[14] Heymann F.J. (1969). High‐speed impact between a liquid drop and a solid surface. J. Appl. Phys..

[15] Visser C.W., Frommhold P.E., Wildeman S., Mettin R., Lohse D., Sun C. (2015). Dynamics of high-speed micro-drop impact: numerical simulations and experiments at frame-to-frame times below 100 ns. Soft Matter.

[16] Mehdizadeh N.Z., Chandra S., Mostaghimi J. (2004). Formation of fingers around the edges of a drop hitting a metal plate with high velocity. J. Fluid Mech..

[17] Field J.E., Dear J.P., Ogren J.E. (1989). The effects of target compliance on liquid drop impact. J. Appl. Phys..

[18] Gonzalez-Avila S.R., Zeng Q., Ohl C.-D. (2024). Pressure and wall shear stress from high-speed droplet impact. Int. J. Multiph. Flow.

[19] Nykteri G., Koukouvinis P., Gonzalez Avila S.R., Ohl C.-D., Gavaises M. (2020). A Σ-ϒ two-fluid model with dynamic local topology detection: Application to high-speed droplet impact. J. Comput. Phys..

[20] Field J.E., Camus J.-J., Tinguely M., Obreschkow D., Farhat M. (2012). Cavitation in impacted drops and jets and the effect on erosion damage thresholds. Wear.

[21] Kondo T., Ando K. (2016). One-way-coupling simulation of cavitation accompanied by high-speed droplet impact. Phys. Fluids.

[22] Tretola G., Vogiatzaki K. (2022). Unveiling the dynamics of ultra high velocity droplet impact on solid surfaces. Sci. Rep..

[23] Biasiori-Poulanges L., Schmidmayer K. (2023). A phenomenological analysis of droplet shock-induced cavitation using a multiphase modeling approach. Phys. Fluids.

[24] Obreschkow D., Dorsaz N., Kobel P., de Bosset A., Tinguely M., Field J., Farhat M. (2011). Confined shocks inside isolated liquid volumes: a new path of erosion?. Phys. Fluids.

[25] Wu Z. (2018). Drop “impact” on an airfoil surface. Adv. Colloid Interface Sci..

[26] Xu S., Fan W., Wu W., Wen H., Wang B. (2023). Analysis of wave converging phenomena inside the shocked two-dimensional cylindrical water column. J. Fluid Mech..

[27] Liu Y., Chu X., Yang G., Weigand B. (2024). Simulation and analytical modeling of high-speed droplet impact onto a surface. Phys. Fluids.

[28] Niu Y.-Y., Wang H.-W. (2016). Simulations of the shock waves and cavitation bubbles during a three-dimensional high-speed droplet impingement based on a two-fluid model. Comput. Fluids.

[29] Hao H., Chen H., Xu S., Charalambides M.N., Sergis A., Taylor A.M.K.P., Chen H., Wang B., Hardalupas Y. (2026). Hydrodynamic and material response to droplet impact on coated turbine blades: implications for cavitation erosion. J. Fluid Mech..

[30] Forehand R.W., Nguyen K.C., Anderson C.J., Shannon R., Grace S.M., Kinzel M.P. (2023). A numerical assessment of shock–droplet interaction modeling including cavitation. Phys. Fluids.

[31] Biasiori-Poulanges L., El-Rabii H. (2021). Shock-induced cavitation and wavefront analysis inside a water droplet. Phys. Fluids.

[32] Sembian S., Liverts M., Tillmark N., Apazidis N. (2016). Plane shock wave interaction with a cylindrical water column. Phys. Fluids.

[33] Thoroddsen S.T., Takehara K., Etoh T.G. (2010). Bubble entrapment through topological change. Phys. Fluids.

[34] Thoroddsen S.T., Etoh T.G., Takehara K., Ootsuka N., Hatsuki Y. (2005). The air bubble entrapped under a drop impacting on a solid surface. J. Fluid Mech..

[35] Langley K.R., Castrejón-Pita A.A., Thoroddsen S.T. (2020). Droplet impacts onto soft solids entrap more air. Soft Matter.

[36] Lee J.S., Weon B.M., Je J.H., Fezzaa K. (2012). How does an Air Film Evolve into a Bubble during Drop Impact?. Phys. Rev. Lett..

[37] Liu Y., Tan P., Xu L. (2013). Compressible air entrapment in high-speed drop impacts on solid surfaces. J. Fluid Mech..

[38] Foltyn P., Rihm L.K., Ribeiro D., Silva A., Weigand B. (2021). Measurement of the lamella thickness during droplet impact onto differently wettable smooth surfaces using an extension of the LASER Pattern Shift Method with naturally occurring patterns. Rev. Sci. Instrum..

[39] Hicks P.D., Purvis R. (2010). Air cushioning and bubble entrapment in three-dimensional droplet impacts. J. Fluid Mech..

[40] Hicks P.D., Purvis R. (2013). Liquid–solid impacts with compressible gas cushioning. J. Fluid Mech..

[41] Van Der Veen R.C.A., Tran T., Lohse D., Sun C. (2012). Direct measurements of air layer profiles under impacting droplets using high-speed color interferometry. Phys. Rev. E.

[42] Haller K.K., Ventikos Y., Poulikakos D., Monkewitz P. (2002). Computational study of high-speed liquid droplet impact. J. Appl. Phys..

[43] Haller K.K., Poulikakos D., Ventikos Y., Monkewitz P. (2003). Shock wave formation in droplet impact on a rigid surface: lateral liquid motion and multiple wave structure in the contact line region. J. Fluid Mech..

[44] Attané P., Girard F., Morin V. (2007). An energy balance approach of the dynamics of drop impact on a solid surface. Phys. Fluids.

[45] Eggers J., Fontelos M.A., Josserand C., Zaleski S. (2010). Drop dynamics after impact on a solid wall: Theory and simulations. Phys. Fluids.

[46] Riboux G., Gordillo J.M. (2014). Experiments of Drops Impacting a Smooth Solid Surface: a Model of the critical Impact speed for Drop Splashing. Phys. Rev. Lett..

[47] Kondo T., Ando K. (2019). Simulation of high-speed droplet impact against a dry/wet rigid wall for understanding the mechanism of liquid jet cleaning. Phys. Fluids.

[48] Tatekura Y., Watanabe M., Kobayashi K., Sanada T. (2018). Pressure generated at the instant of impact between a liquid droplet and solid surface. R. Soc. Open Sci..

[49] G. Nykteri, P. Koukouvinis, S.R.G. Avila, C.-D. Ohl, M. Gavaises, Numerical investigation of high-speed droplet impact using a multiscale two-fluid approach, (2019).

[50] Lukasik G., Rogers J., Kota K.R., Bowersox R.D., Lacy T.E., Kulatilaka W.D. (2021). AIAA SCITECH 2022 Forum.

[51] Sun T.-P., Álvarez-Novoa F., Andrade K., Gutiérrez P., Gordillo L., Cheng X. (2022). Stress distribution and surface shock wave of drop impact. Nat. Commun..

[52] Engel O.G. (1960). Note on particle velocity in collisions between liquid drops and solids. J. RES. NATL. BUR. STAN. SECT. a..

[53] van Dam D.B., Le Clerc C. (2004). Experimental study of the impact of an ink-jet printed droplet on a solid substrate. Phys. Fluids.

[54] Weller H.G., Tabor G., Jasak H., Fureby C. (1998). A tensorial approach to computational continuum mechanics using object-oriented techniques. Comput. Phys..

[55] Brackbill J.U., Kothe D.B., Zemach C. (1992). A continuum method for modeling surface tension. J. Comput. Phys..

[56] Zeng Q., Gonzalez-Avila S.R., Ohl C.-D. (2020). Splitting and jetting of cavitation bubbles in thin gaps. J. Fluid Mech..

[57] Macdonald J.R. (1969). Review of some Experimental and Analytical Equations of State. Rev. Mod. Phys..

[58] Zeng Q., An H., Ohl C.-D. (2022). Wall shear stress from jetting cavitation bubbles: influence of the stand-off distance and liquid viscosity. J. Fluid Mech..

[59] Denner F. (2021). The Gilmore-NASG model to predict single-bubble cavitation in compressible liquids. Ultrason. Sonochem..

[60] Reese H., Ohl C.-D., Rosselló J.M. (2024). Cavitation and jetting from shock wave refocusing near convex liquid surfaces. Int. J. Multiph. Flow.

[61] Horvat D., Agrež V., Požar T., Starman B., Halilovič M., Petkovšek R. (2022). Laser-induced shock-wave-expanded nanobubbles in spherical geometry. Ultrason. Sonochem..

[62] Požar T., Agrež V., Petkovšek R. (2021). Laser-induced cavitation bubbles and shock waves in water near a concave surface. Ultrason. Sonochem..

[63] Hong S., Son G. (2022). Numerical modelling of acoustic cavitation threshold in water with non-condensable bubble nuclei. Ultrason. Sonochem..

[64] Herbert E., Balibar S., Caupin F. (2006). Cavitation pressure in water. Phys. Rev. E.

